# Effect of gibberellic acid (GA), indole acetic acid (IAA) and benzylaminopurine (BAP) on the synthesis of essential oils and the isomerization of methyl chavicol and trans-anethole in *Ocimum gratissimum* L

**DOI:** 10.1186/2193-1801-3-321

**Published:** 2014-06-26

**Authors:** Zakaria Hazzoumi, Youssef Moustakime, Khalid Amrani Joutei

**Affiliations:** Laboratory of Bioactive Molecules: Structure and Function, Faculty of Science and Technology Fez, B.P. 2202, Route d’Imouzzer, FEZ Morocco

**Keywords:** *O. gratissimum* L, Plant growth regulators, Essential oil, Methyl chavicol, Trans-anethole

## Abstract

Basil (*O. gratissimum* L) is a aromatic and medicinal plant widely used in traditional medicine in Morocco. The aim of this work was to study the effect of three plant growth regulators gibberellic acid (GA), indole 3-acetic acid (IAA) and benzylaminopurine (BAP) on the content and composition of essential oils of this plant, especially on the main compound (methyl chavicol) and its isomer (the trans-anethole).

The results showed a wide variation on yield, content and range of the molecule constituent of oil, with a balance of appearances and/or disappearances of a few molecules.

GA caused a slight decrease in the oil yield (0.2%), but it increased the diversity of compounds (17 molecules) with the appearance of four new compounds (naphthalene, camphor, germacrene-D, and ledene) and disappearance of (β cedrene, azulene). This variation also caused a very important decrease in the main compound (methyl chavicol) and increases its isomer (trans-anethole).

IAA and BAP caused an increase in the yield of essential oil (0.30% and 0.32% respectively) without much influence on the main compounds, but with some change in the composition such as the appearance of (germacrene-D) and the disappearance of (aristolene).

## Background

The pharmaceutical and cosmetic industries rely on the purity and heterogeneity of essential oils to reply to the needs and requirements of the market, in the aim to have an effective biological activity (antifungal, antibacterial) or to improve the quality of a cosmetic product.

The content and composition of essential oils are highly influenced by external factors and cultural practices. In Morocco, the *Ocimum* genus and specifically *O. gratissimum* L species is widely used in traditional medicine. Essential oils of this species occupy a very important place in the international oil market.

The antibacterial action of basil essential oil increases with the increase in levels of methyl chavicol (Pessoa et al. 2002 Kishore Dubey et al. 2000). The heterogeneity of oils may also be obtained by the synergistic effect of the different compounds (Ngassoum et al. 2003).

Salah el deen Et (1996) studied the influence of GA, IAA and kinetin on yield and composition of essential oils of *O. basilicum*. They showed that the GA leads to the decrease in essential oil yield while kinetin and IAA increased the yield. This change is accompanied by a decrease in levels of the main compound (methyl chavicol) for all treatments (from 75.16% in the control to 74.1%, 73.2% and 70.7% in kinetin, IAA and GA respectively). The same observation was made by Fraternale et al. (2003) who found that spraying plants *Thymus mastichina* by cytokinin caused an increase in the concentration of essential oils.

Prins et al. (2010), Gershenzon (1994) and Erbelgin et al. (2006) attributed this variation in yield directly to the number of glandular hairs: GA leads to a decrease in the number of these structures whereas treatment with BAP (benzylaminopurine) increases the number of glands in the leaves in several species (*Lavandula dentata*, *Thymus mastichina* and *Picea abies*).

Kim et al. (2006) and Li et al. (2007) showed that the levels of monoterpenes and particularly methyl chavicol change in the essential oil of basil (*O. basilicum* L) after spraying methyl jasmonate. Rodriguez-Saona et al. (2001) also found that GA changes the contents of monoterpenes in *Gossypium hirsutum* L.

These authors have attributed this variation to the genes responsible for regulating the metabolic pathway of monoterpenes. This regulation is due to the change in the enzyme catalytic of these reactions such as the lipoxygenase (LOX), cinnamic acid 4-hydroxylase (C4H), prephenate dehydrogenase (PDH), polyphenol oxidase (PPO), phosphatase acid (APase), and pentatricopeptide repeat (PPR). These enzymes play an essential role in the synthesis of secondary metabolites.

Moreover, Lewinsohn et al. (2000) distinguished two chemotypes in *O.basilicum* L, methyl chavicol (or estragol) and the methyl eugenol. The isomerization between these two molecules is mainly due to the complex enzyme O-methyl transferase.

**Figure 1:**
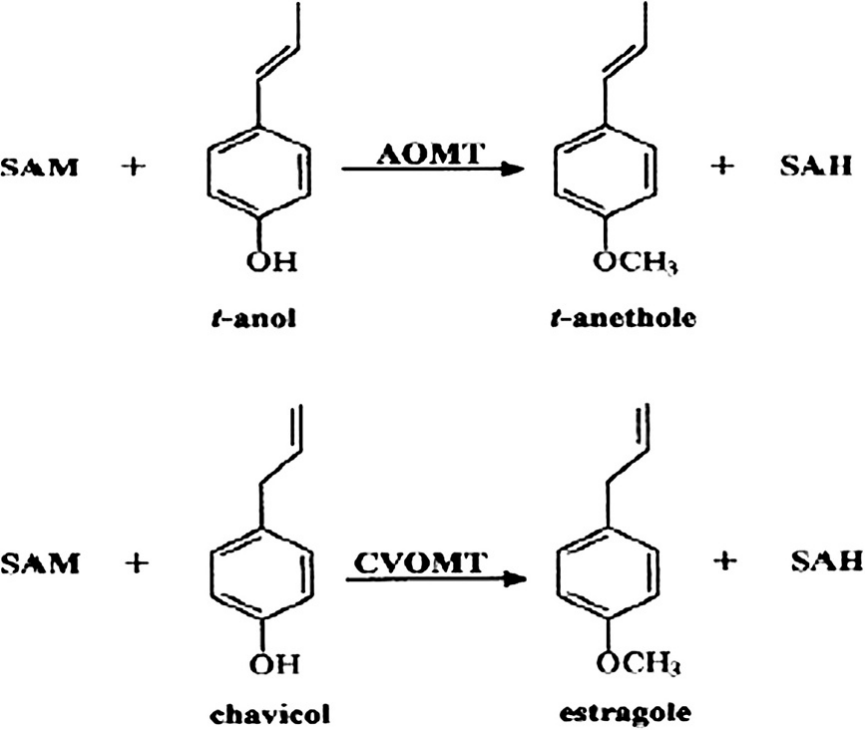
**O-methyltransferase activity in** ***F.vulgare*** **According to Gross et al. (** 2002 **).**The O-methylation of t-anol to t-anethole with SAM O-methyl transferase (AOMT) and methylation of chavicol to methyl chavicol with chavicol (COMT). SAM: S-adenosyl-L-méthionine, SAH: S-adenosyl-homocysteine.

In bitter fennel (*Foeniculum vulgare*) (Figure 1), this enzyme is present in tissues with bispecificity, the first one is called COMT which gives the methyl chavicol from chavicol, the second one is called AOMT that gives the trans-anethole from the trans-anol (Gross et al. 2002).

From those bibliographic data, we are interested in studying the influence of three plant growth regulators GA, IAA and BAP on the yield and the composition of essential oils of *O.gratissimum* L in order to provide useful information to pharmaceutical or cosmetic industries by changing these compositions. On the other hand, to investigate the influence of these plant growth regulators on chavicol isomerization of m-chavicol and t-anethole.

## Materials and methods

### Pregermination of seeds and culturing of basil plantlets

The seeds of basil (*O. gratissimum* L) were disinfected by a passage in ethanol 95% (30 sec to 1 min), then immersed in a solution of mercury hypochlorite (1%) for 3 to 4 minutes and soaked in sterile distilled water in Petri dishes. The seeds are then placed in an incubator at 26°C to germinate. After germination, the seedlings are transplanted into plastic pots (3 kg capacity) at a rate of 50 to 60 per pot and placed in a growth chamber at a temperature between 25 and 27°C. During growth, the plants are sprayed with 70 ppm of each plant growth regulators (GA, IAA and BAP), the first spray was applied two weeks after the transplantation, the other two sprays were applied with an interval of 20 days.

### Extraction of essential oils

100 g of dried aerial parts of *O. gratissimum* were submitted to hydrodistillation with a Clevenger-type apparatus (Clevenger 1928) and extracted with 2L of water for 180 min (until no more essential oil was obtained). The essential oil was collected, dried under anhydrous sodium sulphate and stored at 4˚C until analysed.

The essential oil yield is given by the following formula **YEO (ml/100 g Dm) = (V/Dm × 100) ± (ΔV/ Dm × 100)**

Y.E.O: essential oil yield of dry matter

V: volume of essential oils collected (ml)

ΔV: reading error

Dm: dry plant mass (g)

### GC/MS analysis

Gas chromatography (GC) analyses were performed on a Hewlett-Packard (HP 6890) gas chromatograph (FID), equipped with a 5% phenyl methyl silicone HP-5 capillary column (30 m × 0.25 mm × film thickness 0.25 μm). The temperature was programmed from 50°C after 5 min initial hold to 200°C at 4°C/min. Gas c hromatography conditions were as follows: N2 as carrier gas (1.8 ml/min); split mode was used (Flow: 72.1 ml/min, ratio: 1/50); temperature of injector and detector was 250°C, Fin al hold time was 48 min. The machine was led by a computer system type ″HP ChemStation″, managing the functioning of the machine and allowing to follow the evolution of chromatographic analyses. Diluted samples (1/20 in methanol) of 1 μl were injected manually.

GC/MS qualitative analyses were performed on a Hewlett-Packard equipped with a HP-5MS (Crosslinked 5% PHME Siloxane) capillary column (30 m × 0.25 mm i.d, 0.25 μm film thickness) and coupled with a mass spectrometer (HP 5973). The temperature was programmed from 50 to 250°C at 2°C/min. The carrier gas was He (1.5 ml/min) and split mode was used (Flow: 112 ml/min, ratio: 1/74.7). The different compounds were confirmed by reference to their MS identities (Library of NIST/EPA/NIH MASS SPECTRAL LIBRARY Version 2.0, build Jul 1 2002). MS operating parameters were: ionization voltages 70eV, ion source temperature 230°C, scan mass range 35–450 amu.

## Results

Table 1 shows the contents of essential oils from plants *O. gratissimum* and their chemical compositions. It is noted that the control plants showed a yield of 0.22% and were characterized by a large diversity of molecules with the dominance of methyl chavicol (85.26%) and its isomer trans-anethole (4.25%). Furthermore, it is noticed that these two molecules are the main compounds whatever treatment used, their content is the main part with 89.51% of the entire oil.

**Table 1:** **Influence of (GA, IAA and BAP) on the content and composition of essential oils of** ***O. gratisimum*** **L**

Traetments	Control	GA	IAA	BAP
Oil content (%)	0.22	0.20	0.30	0.32
**RT Components**	**Peak area**			
11.05 Eucalyptol	2.02	0.52	2.71	2.37
15.06 α- campholene	0.20	0.72	0.37	0.27
17.12 **methyl-chavicol**	**85.26**	**58.31**	**86.97**	**84.09**
20.07 **trans anethole**	**4.25**	**12.30**	**2.74**	**4.26**
21.76 Cis anethole	0.08	0.14	0.31	0.34
24.31 Caryophyllene	0.21	0.14	0.35	0.51
24.65 Calarene	0.50	0.16	0.52	0.59
24.93 α-Longipinene	0.82	1.10	0.71	1.27
25.33 α-Caryophyllene	0.52	2.04	0.34	0.44
26.25 Aristolene	0.73	0.61	**-**	**-**
26.17 Germacrene-D	-	3.19	0.89	1.34
26.67 β Cedrene	0.21	-	0.13	0.14
27.35 aromadendrene	0.21	1.37	0.27	**-**
30.20 Cubenol	0.19	2.23	0.18	0.16
30.80 Cadinene	0.78	3.83	1.35	1.63
31.01 Naphthalene	**-**	0.84	**-**	**-**
31.19 camphor	**-**	2.74	**-**	**-**
31.35 Azulene	0.44	**-**	0.68	0.74
37.66 Ledene	**-**	0.59	**-**	**-**

However, we find that the GA does not have a great influence on yield (0.2%), while the IAA and BAP provide a significant increase in the amount of essential oil that can reach 0.3%.

**Figure 2:**
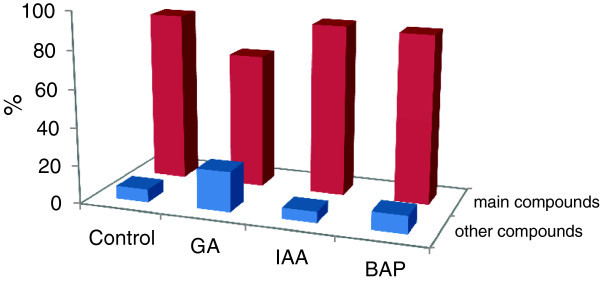
**Contents of main compounds (methyl chavicol and trans-anethole) and other compounds after treatment (GA, IAA and BAP) on plants** ***O. gratisimum*** **L.**

The use of plant growth regulators shows that the predominant compounds (methyl chavicol and trans-anethole) and other compounds (eucalyptol, azulene ......) undergo some changes with balance decrease and increase depending on the nature of the treatment (Table 1, Figure 2):

GA causes a reduction of the levels of main compounds with a value that reached 70.6% due to a dramatic decrease of Methyl chavicol with 58.31%. Whereas, the trans-anethole increases to 12.30%. Moreover, the level of other compounds increased (21.37%) with the appearance of four compounds (germacrene-D, Naphthalene, Juniper camphor, Ledene) and disappearance of two molecules (β cedrene, Azulene). We also note a very significant change in the levels of pre-existing molecules. Cadinene content increases by a factor of 4 (0.78% to 3.83%), the content Cubenol increased 11 times (from 0.19% to 2.23%) and the content aromadendrene increases by a factor of 6 (0.21% to 1.37%). Whereas, the content of Eucalyptol was reduced by a factor of 4 (2.02% 0.52%).

Regarding the IAA, Figure 2 shows that the EO of the plants treated by this auxin do not exhibit a significant change in main compounds since the sum of these molecules is substantially the same as the control with a slight increase in methyl chavicol and slight decrease in trans anethole (Table 1). The sum of other compounds is also identical (6.13%), but we note the appearance of germacrene-D and the disappearance of Aristolene.

The 3rd treatment which relates to the use of BAP shows the same results as the IAA, the only difference was represented by the disappearance of aromadendrene (Table 1).

## Discussion

### Influence of plant growth regulators on the essential oils of *O. gratisimum*L

The yield of essential oils of *O. gratisimum* L is around 0.22%, similar to that found in Egyptian *O. basilicum* L (0.213%) (Salah el deen Et 1996), but it is lower than that found in Turkish and Iranian basil (Telci et al. 2006; Ebrahim 2006), which value were close to 0.5%.

Moreover, *O. gratisimum* L is characterized by the methyl chavicol as a main compound with a value of 85.26%, which is higher than Egyptian, Iranian and the Middle East basil (Salah el deen Et 1996; Ebrahim 2006; Werker et al. 1993) they vary from 48% to 75%. But, this value is almost similar to the *O. basilicum* L of Comoros Islands whose contents vary between 74% and 84% (Randriamiharisoa et al. 1986).

GA causes a decrease in the yield of essential oils and a variation in the composition of these oils. These results are confirmed by those of Salah el deen Et (1996) who showed that the GA application on plants basil caused a reduction in yield which is accompanied by a decrease in the content of methyl chavicol and an increase of Eucalyptol. These results are also found in other plants such as *Thymus vulgaris* L., which show an increase in the yield of essential oils after GA application (Reda et al. 2005).

On the other side, Poyh and Ono (2007) found an increase in the yield of EO in *Salvia officinalis* L plants after spraying with GA (100 mg/L), this increase goes with a decrease of main compound α-thujone.

Moreover, IAA leads to an increase in the yield of *O. gratissimum* L essential oils which passes from 0.22% to 0.3%, with no significant change neither in the content nor the main compounds composition of essential oils. These results are the same as those found in the literature. Salah el deen Et (1996) showed that the application of the IAA increases slightly the yield of *O.basilicum* L oils, this variation goes with small change in methyl chavicol. The same observation was made on Lemon balm (*Melissa officinalis* L) (Shukla and Farooqi 1990) and *Thymus vulgaris* L (Affonso et al. 2009). On the other side, they have noticed a very large increase in the main compound (315% Thymol) without any variation the quality of the essential oils.

Regarding the BAP, it is found that this phytohormone acts like IAA with an increase in the yield of essential oils and without any variation in methyl chavicol content. This quantitative variation has been detected in several species *Lavandula dentata* L (Oudin et al. 2007), *Thymus mastichina* L (Fraternale et al. 2003), *Mentha piperita*, *M. spicata*, *M. suaveolens*, *Salvia officinalis*, *Lavandula vera* (El-Keltawi and Croteau 1987), *Cymbopogon citratus* L. (Craveiro et al. 1989).

We can explain this change in the yield of EO in *O. gratissimum* plants after treatment with phytohormone on the basis of changes in the leaf area and the density of the glandular hairs of the leaves. Scravoni et al. (2006) found an increase in dry weight of *Mentha piperita* L plant treated with BAP (50 mg/L). Furthermore, Fraternale et al. (2003) showed that the spraying with BAP on *Thymus mastichina* plants causes an increase of the density of the glandular hairs comparing to control plants.

**Figure 3:**
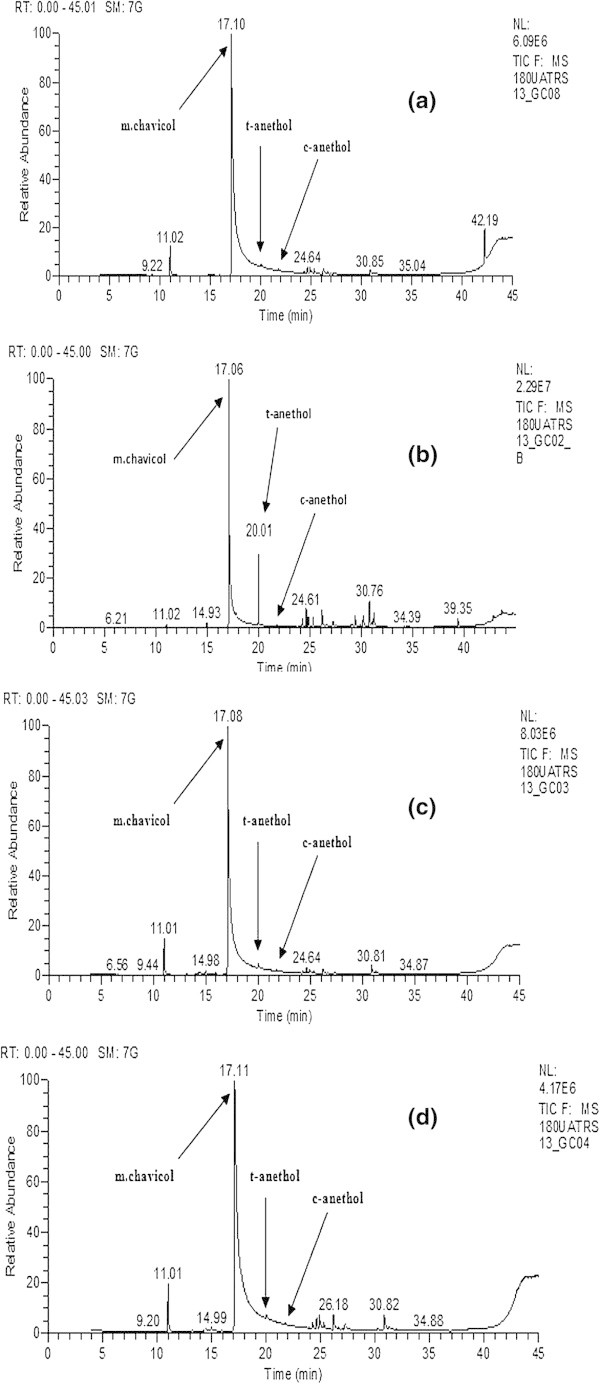
**Chromatograms of essential oils extracted from** ***O. gratissimum*** **L plants: (a) control, (b) GA, (c) IAA and (d) BAP.**

From the results of Table 1 and Figure 3, we found that the increase in levels of main compounds (methyl chavicole and trans-anethole) is accompanied by a decrease in other compounds and vice versa, especially in plants treated with GA that show a significant decrease in main compounds and an increase in other compounds. This can be explained by the metabolic pathways of plants that change with the application of the treatment, causing the appearance of new molecules and/or the disappearance of existing ones. These findings were confirmed by the works of Kim et al. (2006), Li et al. (2007), and Erbelgin et al. (2006) who reported that monoterpenes *O. baslicum* and *Lavendula dentata* are highly influenced by plant growth substance treatments due to the genes regulation which cause an increase in enzyme numbers related to the metabolic pathways of these compounds.

El-Keltawi and Croteau (1987) suggested that cytokinins stimulate the metabolism and accumulation of essential oils, specifically monoterpenes in *Mentha piperita* L, and *Salvia officinalis* L.

### Influence of plant growth regulators on the two isomers: methyl chavicol and trans-anethole

Essential oils of our plants (*O. gratissimum*) are characterized by methyl chavicol as chemotype, with the presence of its isomer (t-anethole). These two isomers are different only in the double bond of the propenyl chain, and we can find this natural isomer in many EO of plants, tarragon (*Artemisia dracunculus* L), basil (*O. basilicum* L) and (*Foeniculum vulgare*) (Gross et al. 2002) but with different contents. This difference is mainly due to the enzyme complex (O-methyl transferase), which uses chavicol and trans-anol as substrate to produce methyl chavicol and trans-anethole respectively (Figure 1) (Lewinsohn et al. 2000; Gross et al. 2002).

Furthermore our results show that these two isomers progress in opposite ways: an increase of a molecule causes a decrease of its isomer and vice versa (Table 1).

**Figure 4:**
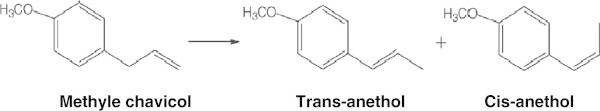
**Isomerization of chavicol to anethole according to D. Kishore (** 2006 **).**

This evolution of the content of the two isomers and the absence of trans-anol suggest that in *O. gratissimum* L, the synthesis pathway of trans-anethole pass through direct isomerization of methyl chavicol (Figure 4) probably through an enzyme complex and not by the transformation of trans anol as already suggested by Gross et al. (2002). In vitro catalysis of this reaction is possible and widely used in the food industry (Kishore 2006; Sharma et al. 2005). Low levels of cis anethole found in our extracts can be attributed to the instability of the cis configuration.

In plants treated with GA (Figure 3 and Table 1), the significant increase in levels of t-anethole also suggest that this phytohormone acts on the enzyme complex responsible for the isomerization of m-chavicol to t-anethole.

## Conclusion

In this study we are trying to investigate the effects of different plant growth regulators on yield, composition and content of essential oils in order to have the opportunity to improve and/or change the composition of some molecules having economical interest (pharmacological or cosmetic).

Essential oils of *O. gratissimum* L are characterized by the m-chavicol and its isomer t-anethole as main compounds. Furthermore, the absence of t-anol suggests that there is a direct isomerization between these two molecules.

The GA application leads to a decrease in the levels of m-chavicol and an increase in t-anethole. We notice the emergence of other molecules (germacrene-D, Naphthalene, Ledene and comphore).

The applications of IAA and BAP have the effect of increasing the yield of essential oils without remarkable qualitative change.

Further research such as the purification of enzymes in these plants, as well as the isolation and analysis of their genes will help to understand the mechanism of change in monoterpene biosynthetic pathways specifically in these plants and to check the influence of GA on the isomerization of Methyl chavicol to trans anethole.

## Abbreviations

m-chavicol: Methyle chavicol
t-anethole: Trans-anethol
GA: Gibberellic acid
IAA: Indole 3-acetic acid
BAP: Benzylaminopurine
COMT: Chavicol O-methyl transferase
AOMT: Anethol O-methyl transferase
EO: Essential oil.

## References

[CR1] Affonso VR, Bizzo HR, Lage CLS, Sato A (2009). Influence of growth regulators in biomass production and volatile profile of in vitro Plantlets of *Thymus vulgaris* L. J Agric Food Chem.

[CR2] Clevenger JF (1928). Determination of volatile oil. J Ann Pharm Assoc.

[CR3] Craveiro A, Barreira ES, Rabi J, Dagnino D (1989). Estudo sobre o efeito de citocininas na biossíntese de monoterpenos.

[CR4] Dubey K, Tiwari TN, Danielle M, Hary A, Jean-Pierre C (2000). Antifungal properties of *Ocimum gratissimum* essential oil (ethyl cinnamate chemotype). Fitoterapia.

[CR5] Ebrahim S (2006). Analysis of the essential oils of two cultivated basil (*Ocimum basilicum* L.). From Iran Daru.

[CR6] El-Keltawi NE, Croteau R (1987). Influence of foliar applied cytokinins on growth and essential oil content of several members of the lamiaceae. Phytochemistry.

[CR7] Erbelgin N, Krokene P, Christiansen E, Gazmend Z, Gershenzon J (2006). Exogenous application of methyl jasmonate elicits defenses in Norway spruce (*Picea abies*) and reduces host colonization by the bark beetle Ips typographus. Oecologia.

[CR8] Fraternale D, Giamperi L, Ricci D, Rocchi MBL, Guidi L, Epifano F, Marcotullio FC (2003). The effect of triacontanol on micropropagation and on secretory system of *Thymus mastichina*. Plant Cell Tissue Organ Cult.

[CR9] Gershenzon J (1994). Metabolic costs of terpenoid accumulation in higher plants. J Chem Ecol.

[CR10] Gross M, Jacob F, Nativ D, Olga L, Yael C, Einat B, Uzi R, Eli P, Efraim L (2002). Biosynthesis of estragole and t-anethole in bitter fennel (Foeniculum vulgare Mill. var. vulgare) chemotypes. Changes in SAM:phenylpropene O-methyltransferase activities during development. Plant Sci.

[CR11] Kim H-J, Chen F, Chen F, Rajapakse NC (2006). Effect of methyl jasmonate on secondary metabolites of sweet basil (*Ocimum basilicum* L.). J Agric Food Chem.

[CR12] Kishore D (2006). Srinivasan Kannan. Journal of Molecular Catalysis A.

[CR13] Lewinsohn E, Ziv-Raz I, Dudai N, Tadmor Y, Lastochkin E, Olga Larkov A, David C, Uzi R, Eli P, Eran P, Yuval S (2000). Biosynthesis of estragole and methyl-eugenol in sweet basil (Ocimum basilicum L), Developmental and chemotypic association of allylphenol O-methyltransferase activities. Plant Sci.

[CR14] Li Z, Wang X, Chen F, Kim H-J (2007). Chemical changes and over expressed genes in sweet basil (*Ocimum basilicum* L.) upon methyl jasmonate treatment. J Agri. Food Chem.

[CR15] Ngassoum MB, Essia-Ngang JJ, Tatsadjieu LN, Jirovetz L, Buchbauerc G, Adjoudji O (2003). Antimicrobial study of essential oils of *Ocimum gratissimum* leaves and *Zanthoxylum xanthoxyloides* fruits from Cameroon. Fitoterapia.

[CR16] Oudin A, Mahroug S, Courdavault V, Hervouet N, Zelwer C, Rodríguez- Concepción M, St-Pierre B, Burlat V (2007). Spatial distribution and hormonal regulation of gene products from methyl erythritol phosphate and monoterpene-secoiridoid pathways in *Catharanthus roseus*. Plant Mol Biol.

[CR17] Pessoa LM, Morais SM, Bevilaqua CML, Luciano JHS (2002). Anthelmintic activity of essential oil of *Ocimum gratissimum* Linn. and eugenol against Haemonchus contortus. Vet Parasitol.

[CR18] Poyh JÁ, Ono EO (2007). Efeito do ácido giberélico na composição do oleo essencial de *Salvia officinalis* L. Publ UEPG Biol Health Sci.

[CR19] Prins CL, Vieira IJC, Freitas Braz SP (2010). Growth regulators and essential oil production. J Plant Physiol.

[CR20] Randriamiharisoa R, Gaydou EM, Banchini JP, Ravelojaona G, Vernin G (1986). Etude de la variation, de la composition chimique et classification des huiles essentielles de basilic de MADAGASCAR. Science et Aliments.

[CR21] Reda F, Abdel Rahim EA, El Baroty GSA, Ayad HS (2005). Response of essential oils, phenolic components and polyphenol oxidase activity of thyme (*Thymus vulgaris L*.) to some bioregulators and vitamins. J Agr Biol.

[CR22] Rodriguez-Saona C, Crafts-Brandner SJ, Paré PW, Henneberry TJ (2001). Exogenous methyl jasmonate induces volate emissions in cotton plants. J Chem Ecol.

[CR23] Salah el deen Et M (1996). Mahmoud Response of Growth and essential oil content of sweet Basil (*Ocimum basilicum* L) To some naturel hormones, proceeding int .symp. Medicinal and aromatics plants.Eds.L.E Craker, L.Nolan, K.shetty.acta hort. H26,ISHS.

[CR24] Scravoni J, Vasconcellos MC, Valmorbida J, Ferri AF, Marques MOM, Ono EO, Rodrigues JD (2006). Rendimento e composição química do óleo essencial de *Mentha piperita* L. submetida a aplicações de giberelina e citocinina. Rev Bras Pl Med.

[CR25] Sharma SK, Srivastava VK, Pandya PH, Raksh V (2005). Jasra Solvent-free isomerization of methyl chavicol to trans-anethole using transition metal complexes as catalysts. Catalysis Communications.

[CR26] Shukla A, Farooqi AHA (1990). Utilization of plant growth regulators in aromatic plant production. Curr Res Med Arom Plants.

[CR27] Telci I, Emine B, Gungor Y, Betul A (2006). Variability in essential oil composition of Turkish basils (*Ocimum basilicum* L.). Biochem Syst Ecol.

[CR28] Werker E, Putievsky E, Ravid U, Dudai N, Katzir I (1993). Glandular hairs and essential oil in developing leaves of *ocimum basilicum L* (Lamiaceae). Ann Bot.

